# Uterine artery embolization with highly compressible calibrated microspheres for the treatment of uterine fibroids

**DOI:** 10.1590/0100-3984.2021.0123

**Published:** 2022

**Authors:** Denis Szejnfeld, Rômulo Florêncio Tristão Santos, Antonio Massamitsu Kambara, Marcelo Bueno de Oliveira Colli, Felipe Nasser, Mauricio de Sena Martins, Suzan Menasce Goldman

**Affiliations:** 1 Escola Paulista de Medicina da Universidade Federal de São Paulo (EPM-Unifesp), São Paulo, SP, Brazil.; 2 Instituto Dante Pazzanese de Cardiologia, São Paulo, SP, Brazil.; 3 Hospital Santa Marcelina, São Paulo, SP, Brazil.; 4 Hospital Israelita Albert Einstein, São Paulo, SP, Brazil.; 5 Hospital Maternidade Leonor Mendes de Barros, São Paulo, SP, Brazil.

**Keywords:** Leiomyoma, Uterine artery, Uterine artery embolization, Microspheres, Leiomioma, Artéria uterina, Embolização da artéria uterina, Microesferas

## Abstract

**Objective:**

To evaluate the safety and efficacy of using highly compressible calibrated microspheres in uterine artery embolization (UAE) for the treatment of uterine fibroids.

**Materials and Methods:**

This was a prospective multicenter study. Thirty-two women with symptomatic uterine fibroids were selected for UAE between January 2019 and March 2020. The participants completed the Uterine Fibroid Symptom and Quality of Life (UFS-QOL) questionnaire, underwent contrast-enhanced pelvic magnetic resonance imaging (MRI), and were submitted to anti-Müllerian hormone measurement, subsequently undergoing UAE with Embosoft microspheres. After six months, the patients again completed the UFS-QOL questionnaire and underwent pelvic MRI.

**Results:**

The most common symptoms were abnormal uterine bleeding (in 81.3% of the cases), pelvic pain (in 81.3%), and compression (in 46.9%). Of the 32 patients evaluated, 12 (37.5%) had anemia due to abnormal uterine bleeding. Thirty patients completed the study. Among those patients, we observed median reductions of 21.4% in uterine volume and 15.9% in dominant fibroid volume. We identified no adverse events that could be attributed to the material itself, although there were events attributed to the UAE procedure in general.

**Conclusion:**

For the treatment of uterine fibroids, UAE using Embosoft microspheres shows satisfactory results, providing reductions in uterine and dominant fibroid volumes, with a low rate of adverse events, and improving patient quality of life, as well as demonstrating safety and efficacy.

## Abstract (pt)

**Objetivo:**

Avaliar a eficácia e segurança da embolização da artéria uterina (EAU) com microesferas calibradas de alta compressibilidade no tratamento de miomas uterinos.

**Materiais e Métodos:**

Este foi um estudo prospectivo e multicêntrico. Foram selecionadas 32 mulheres com miomas uterinos sintomáticos para EAU de janeiro de 2019 a março de 2020. As participantes preencheram o questionário Uterine Fibroid Symptom and Quality of Life (UFS-QOL), realizaram ressonância magnética (RM) pélvica com contraste e teste para medição dos hormônios antimüllerianos, seguido de embolização de miomas com microesferas Embosoft. Após seis meses, as pacientes novamente preencheram o UFS-QOL e realizaram RM pélvica.

**Resultados:**

Os sintomas mais relatados foram sangramento uterino anormal (81,3%), dor pélvica (81,3%) e compressão (46,9%). Doze pacientes (37,5%) apresentaram anemia consequente a sangramento uterino anormal. Nas 30 pacientes que completaram o estudo, observou-se redução mediana de 21,4% no volume uterino e 15,9% no volume do mioma dominante. Não foram identificados eventos adversos possivelmente relacionados ao material utilizado, apenas em relação ao procedimento de EAU.

**Conclusão:**

EAU com microesferas Embrosoft mostrou resultados satisfatórios no tratamento de miomas uterinos, com redução dos volumes uterino e do mioma dominante, baixa taxa de eventos adversos e melhora na qualidade de vida, demonstrando segurança e eficácia.

## INTRODUCTION

Uterine artery embolization (UAE) emerged as an alternative to hysterectomy in the 1990s^(1-3)^. The procedure was based on well-established techniques for the treatment of pelvic bleeding related to trauma or gynecologic emergencies, such as postpartum hemorrhage, and later for the treatment of uterine fibroids. Since the initial reports, UAE has become a widely accepted, safe, and effective alternative to surgery^(2-7)^, and it is now the leading minimally invasive treatment for symptomatic uterine fibroids^(8)^.

Initial studies have, for the most part, used polyvinyl alcohol particles as the embolic agent^(9,10)^, demonstrating that the material is highly safe and effective. However, some technical difficulties related to microcatheter occlusion have prompted the use of calibrated spheres, with similar or even superior efficacy^(10)^, notably attributed to the increased compressibility of the spheres, which enables them to pass through the microcatheter more easily and to settle better into the embolized vessels, allowing for more distal and consistent embolization.

Embosoft calibrated microspheres (Scitech Medical, Aparecida de Goiânia, Brazil) were developed using Polifit 70 as a coating material, which allows a level of compressibility of approximately 40% with subsequent reexpansion and return to the original spherical shape without fragmentation or deformation, thus providing more effective embolization.

The present study aimed to evaluate the safety and efficacy of UAE for the treatment of fibroids using Embosoft Polifit 70 microspheres.

## MATERIALS AND METHODS

### Study design

This was a prospective multicenter study. The study was approved by the research ethics committees of the participating institutions and was registered at ClinicalTrials.gov (NCT03535610). All participants gave written informed consent.

The gynecology departments of the participating institutions selected a collective total of 32 women between 18 and 50 years age with symptomatic uterine fibroids for treatment with UAE between January 2019 and March 2020. Once selected, the interventional radiology teams of the institutions invited the women to participate in the study. Patients with contraindications to magnetic resonance imaging (MRI), previous surgical treatment, and recent use of gonadotropin-releasing hormone analogues were excluded.

Participants completed the Uterine Fibroid Symptom and Quality of Life (UFS-QOL) questionnaire^(11)^, underwent contrast-enhanced pelvic MRI, and were submitted to anti-Müllerian hormone measurement. Thereafter, participants underwent fibroid embolization with a well-established standard technique, using Embosoft Polifit 70 microspheres with sizes ranging from 300 µm to 900 µm. The standard microsphere size used in the study was 500-700 µm. However, in cases of adenomyosis, one or two syringes of 300-500 µm microspheres could be used on each side prior to embolization with 500-700 µm microspheres. Initially, up to four syringes (8 mL each) of 500-700 µm microspheres were used in each patient (approximately two per side) and, if additional material was still needed, the microsphere size was increased to 700-900 µm. If four syringes (8 mL) of 700-900 µm microspheres (approximately two per side) had been used and stasis had not been reached, Gelfoam could be used in order to achieve complete embolization.

The standard protocol was bilateral embolization. However, unilateral embolization could be used if one uterine artery was dominant (because the other was tapered and was not irrigating the leiomyoma, because there was a single fibroid with unilateral irrigation, or because there was only a single uterine artery). Embolization was performed up to the endpoint, characterized by the “pruned tree” appearance. At six months after UAE, the participants again completed the UFS-QOL questionnaire and underwent contrast-enhanced pelvic MRI.

The primary outcome measures were the efficacy and safety profile of Embosoft Polifit 70 microspheres in UAE. Secondary outcome measures included the reduction in uterine volume and largest fibroid volume at six months after UAE, as assessed by pelvic MRI, and the impact of embolization on quality of life and ovarian function.

### Data collection and statistical analysis

Data were collected and managed using Research Electronic Data Capture tools^(12)^ and analyzed in R version 2020^(13)^. Descriptive statistics, including means and standard deviations, were used in order to summarize continuous variables. The data were analyzed at two time points: baseline and six months after UAE. Given the small sample size, the Wilcoxon test was used in order to analyze continuous variables. The level of statistical significance was set at *p* ≤ 0.05.

## RESULTS

A total of 32 patients were included in the study, with a mean age of 40.9 years (range, 28-49 years). Of those 32 patients, 17 (53.1%) were mixed-race, nine (28.1%) were white, and six (18.8%) were black. The mean body weight was 77.5 ± 17.2 kg, and the mean body mass index was 29.7 ± 6.2 kg/m^2^. Fifteen patients (46.9%) were using oral hormonal contraceptives. Thirteen patients (40.6%) had no children, and 19 (59.4%) had children. Patient data are shown in Table 1.

**Table 1:** Baseline characteristics of patients undergoing UAE.

Characteristic	(N = 32)
Age (years), mean ± SD (range)	40.91 ± 5.81 (28-49)
Race, n (%)	
White	9 (28.1)
Black	6 (18.8)
Mixed	17 (53.1)
Weight (kg), mean ± SD	77.56 ± 17.27
Oral contraceptive use, n (%)	
Parity, n (%)	15 (46.9)
0	13 (40.6)
≥ 1	19 (59.4)

Eleven patients (34.4%) had regular menstrual cycles. The most commonly reported symptoms were abnormal uterine bleeding (in 81.3%), pelvic pain (in 81.3%), and compression (in 46.9%). Twelve patients (37.5%) had anemia due to abnormal uterine bleeding. Of the 32 patients, 10 (31.3%) had previously undergone pharmacologic fibroid treatment, five (15.6%) had previously undergone hysteroscopy, and one (3.1%) had previously undergone laparoscopy.

The combination of uterine fibroids and adenomyosis was observed in 34.4% of the cases, in the focal form in 18.2% and in the diffuse form in 81.8%. Endometriosis accompanied by fibroids was observed in 6.3% of the cases. The mean number of fibroids seen on MRI was 6 (range, 1-16).

Procedure-related data, such as type of anesthesia, arterial access site, intraprocedural medications, unilateral or bilateral embolization, and microsphere size, are shown in Table 2. Adverse events that occurred immediately after UAE and during follow-up are shown in Table 3.

**Table 2:** Variables related to the UAE procedure.

Variable	(N = 32)^*^
Anesthesia, n (%)	
Spinal	20 (62.5)
Epidural	3 (9.4)
Local	9 (28.1)
Femoral access, n (%)	32 (100)
Intraprocedural medications, n (%)	
Antibiotics	28 (87.5)
Anti-inflammatory drugs	20 (62.5)
Analgesics	28 (87.5)
Bilateral embolization, n (%)	32 (100)
Microsphere size (µm), n (%)	
300-500	3 (9.4)
500-700	23 (71.9)
700-900	6 (18.8)

* In two cases, Gelfoam was required in order to achieve stasis. No complications occurred during any of the procedures.

**Table 3:** Adverse events immediately after UAE and during follow-up

Adverse event	(N = 32)
Immediately after UAE, n (%)	
Nausea	12 (37.5)
Vomiting	9 (28.1)
Pelvic pain	12 (37.5)
Facial rash	1 (3.1)
Blood pressure peak	1 (3.1)
During follow-up, n (%)	
Nausea	1 (3.1)
Pelvic pain	4 (12.5)
Fibroid expulsion	0 (0)
Menorrhagia	2 (6.2)
Hysterectomy	0 (0)

Of the 32 patients, 30 were reevaluated at six months after UAE. Two patients were lost to follow-up: one failed to return to the research center after UAE; and one dropped out of the study to undergo elective hysterectomy at another institution. Among the 30 patients who completed the study, we observed a median reduction of 21.4% in uterine volume and of 15.9% in dominant fibroid volume. Those results are shown in Table 4, together with the anti-Müllerian hormone levels. The UFS-QOL questionnaire results are shown in Table 5.

**Table 4:** Primary outcomes after UAE.

Outcome	Before UAE (n = 30)	Six months after UAE (n = 30)	Reduction (95% CI)	*P*-value
Uterine volume (cm^3^)	302 (252-667.25)	237.5 (159.1-447.98)	-29.80 (-41.37 to -9.85)	0.0003
Dominant fibroid volume (cm^3^)	5.35 (3.55-7.08)	4.15 (2.35-5.5)	-15.94 (-27.63 to -9.17)	0.0035
Anti-Müllerian hormone (ng/mL)	0.55 (0.02-3.02)	0.22 (0.05-0.78)	-0.20 (-0.61 to -0.10)	0.0001

Data are presented as median (interquartile range)

**Table 5:** UFS-QOL questionnaire results.

UFS-QOL scores	Before UAE (n = 30)	Six months after UAE (n = 30)	Difference (95% CI)	*P*-value
Symptom severity*	65.63 (57.03- 80.47)	28.13 (15.63-42.97)	-32.81 (-46.87 to -28.12)	< 0.0001
Concern^†^	32.5 (15.0-50.0)	82.50 (70.0-93.75)	42.50 (25.00-55.00)	< 0.0001
Activities^†^	32.14 (17.86-60.71)	87.50 (71.43-96.43)	46.42 (32.14-67.85)	< 0.0001
Energy/Mood^†^	26.79 (11.61-48.21)	76.79 (60.71-96.43)	48.21 (26.78-67.85)	< 0.0001
Control^†^	22.50 (6.25-45.0)	82.50 (70.0-95.0)	50.00 (32.50-65.00)	< 0.0001
Self-consciousness^†^	16.67 (2.08-31.25)	66.67 (52.08-83.33)	41.66 (33.33-58.33)	< 0.0001
Sexual function^†^	37.5 (0.0-62.5)	100 (53.13-100)	37.50 (12.50-56.25)	0.0001
Total^†^	29.31 (15.09-42.67)	83.19 (64.88-92.89)	41.38 (27.59-63.80)	< 0.0001

Results expressed as median (interquartile range).

* Higher value = greater severity.

† Higher value = better quality of life.

## DISCUSSION

The use of UAE has gained acceptance as an alternative to myomectomy or hysterectomy in women with symptomatic uterine fibroids^(14)^. Many women are satisfied with the results of this procedure, which include improvement of symptoms and a reduction in uterine volume, with preservation of the uterus and of fertility^(7,8)^.

The present study was designed to evaluate the short- and medium-term results of UAE using Embosoft Polifit 70 microspheres. In particular, the safety profile of the microspheres was objectively quantified based on adverse events, volume reductions (of the uterus and dominant fibroid), impact on quality of life, and impact on ovarian function. Safety issues are extremely important when using a new material, and verifying the absence of procedure- and material-related complications is the best way to ensure safety.

In the present study, we found that UAE with Embosoft Polifit 70 microspheres resulted in a uterine volume reduction of 21.4% and dominant fibroid volume reduction of 15.9%. For comparison, Spies et al.^(9)^ and Spies et al.^(10)^ evaluated the same outcomes after UAE with tris-acryl gelatin microspheres, polyvinyl alcohol particles, and spherical polyvinyl alcohol, finding uterine volume reductions of 27.4%, 30.2%, and 16.4%, respectively, and dominant fibroid volume reductions of 39.0%, 42.5%, and 29.6%, respectively. Siskin et al.^(15)^ evaluated uterine volume and dominant fibroid volume after embolization with tris-acryl gelatin microspheres and spherical polyvinyl alcohol, finding a uterine volume reduction of 12.6% and 16.5%, respectively, and a dominant fibroid volume reduction of 18.0% and 26.2%, respectively. Complication rates were similar across all studies^(9,10,15)^. Comparing different embolic agents, Siskin et al.^(15)^ reported percentage reductions in uterine volume and in dominant fibroid volume similar to those found in the present study. However, Spies et al.^(9)^ and Spies et al.^(10)^ reported reductions in uterine and dominant fibroid volumes greater than those observed in our sample.

All operators were very careful to ensure that the endpoint was achieved; that is, that the embolization was complete and stable. Improvement in fibroid-related clinical symptoms after UAE is the most important measure of clinical efficacy^(16)^. In addition to operator skill, the characteristics of the material employed have a direct impact on the level of fibroid devascularization achieved^(16-18)^. One major concern is the recurrence of fibroid growth and symptoms over the long term. To our knowledge, there have been no definitive studies assessing fibroid recurrence, and it therefore remains unknown what the rate would be^(9)^. In the present study, patients were followed for six months after UAE. Future studies could assess the degree of fibroid devascularization and rate of fibroid recurrence over longer periods.

In the present study, we identified no adverse events that could be attributed to the material itself, although there were events attributed to the UAE procedure in general. Although fibroid expulsion after UAE has been reported to occur in 1.7-50.0% of cases^(19)^, that complication was not observed in our sample. Our study has some limitations, including the medium-term clinical follow-up and the relatively small sample size.

It has been well established that UAE produces consistent results in terms of symptom resolution. Although not recommended as a first-line therapy in patients who plan to become pregnant, UAE has a wide range of indications in women of reproductive age, which has generated great interest in its potential impact on female fertility^(20,21)^.

In summary, UAE using Embosoft Polifit 70 microspheres showed satisfactory results for the treatment of uterine fibroids, as well as demonstrating safety and efficacy. The procedure provides a reduction in uterine and dominant fibroid volumes, has a low rate of adverse events, and improves quality of life.
